# Reactive Oxygen Species as a Link between Antioxidant Pathways and Autophagy

**DOI:** 10.1155/2021/5583215

**Published:** 2021-07-21

**Authors:** Dan Li, Zongxian Ding, Kaili Du, Xiangshi Ye, Shixue Cheng

**Affiliations:** Collaborative Innovation Center of Yangtze River Delta Region Green Pharmaceuticals, College of Pharmaceutical Sciences, Zhejiang University of Technology, Hangzhou, China

## Abstract

Reactive oxygen species (ROS) are highly reactive molecules that can oxidize proteins, lipids, and DNA. Under physiological conditions, ROS are mainly generated in the mitochondria during aerobic metabolism. Under pathological conditions, excessive ROS disrupt cellular homeostasis. High levels of ROS result in severe oxidative damage to the cellular machinery. However, a low/mild level of ROS could serve as a signal to trigger cell survival mechanisms. To prevent and cope with oxidative damage to biomolecules, cells have developed various antioxidant and detoxifying mechanisms. Meanwhile, ROS can initiate autophagy, a process of self-clearance, which helps to reduce oxidative damage by engulfing and degrading oxidized substance. This review summarizes the interactions among ROS, autophagy, and antioxidant pathways. The effects of natural phytochemicals on autophagy induction, antioxidation, and dual-function are also discussed.

## 1. Introduction

Reactive oxygen species (ROS) are generally small, short-lived, and highly reactive molecules that are formed by incomplete one-electron reduction of oxygen. ROS are generated by multiple cellular organelles, including mitochondria, peroxisomes, and endoplasmic reticulum [1–3]. ROS can also be produced in Fenton and Haber-Weiss reactions, thymidine catabolism, and polyamine catabolism. Mitochondria are the major source of ROS generation, as a by-product of respiration [4].

Under pathological conditions, dysfunctional mitochondria produced excessive ROS, breaking cellular homeostasis. The process of removal of damaged mitochondria through autophagyis called mitophagy, which is thus critical for maintaining cellular functions [5, 6]. Autophagy and mitophagy are important cellular processes that are responsible for removing excessive ROS and damaged organelles. Cells have also developed various antioxidant and detoxifying mechanisms. So far, there are over 20 redox-sensitive transcription factors, found in human cells [7–9]. In addition, ROS have been identified as a signal molecule in various pathways regulating cell survival and cell death [10, 11].

In response to oxidative stress, autophagy is found to contribute to antioxidant function [12, 13]. Recent studies have shown that ROS play a crucial role in autophagy initiation [14]. On the one hand, stimulating factors such as starvation, pathogens, or death receptors initiate autophagy via ROS [15]. On the other hand, oxidized biomaterials such as damaged mitochondria are targeted by autophagy for lysosomal degradation [6, 16]. Hence, ROS and autophagy constitute a negative feedback mechanism that mitigates oxidative stress and promotes cell survival [17]. However, single treatment with antioxidant or autophagy activator has defects on treating diseases with autophagy dysfunction and antioxidative stress. Studies have been conducted to investigate dual-target treatments that can regulate both antioxidant pathways and autophagy [18].

The purpose of this review is to summarize the molecular mechanisms of ROS signals, autophagy, and redox regulation in health and disease. Furthermore, dual-target phytochemicals based on autophagy and antioxidant regulation are exemplified and discussed.

## 2. ROS and Oxidative Stress

ROS are single-electron reduction products of oxygen that include superoxide anion (O_2_^–^), hydrogen peroxide (H_2_O_2_), and hydroxyl radical (HO^∙^), but also diverse peroxides, such as lipid peroxides, peroxides of proteins, and nucleic acids [4, 19, 20]. ROS attack amino acid residues of proteins, specifically Tyr, Phe, Trp, Met, and Cys, to form carbonyl derivatives and promote intra- and intermolecular crosslinking through the formation of disulfide bonds. Superoxide generates hydroxyl free radicals, which initiate peroxidation of phospholipids [21]. The majority of ROS produced in mitochondria are dependent on the respiratory chain complexes I and III and a variety of enzymes [22]. Complex I (NADH-ubiquinone oxidoreductase, C-I), an integral inner membrane polyprotein complex, is considered to be the most significant source of ROS in mitochondria. But the exact site within the C-I is not clear [23]. Another pathway of ROS generation is the Q cycle in complex III, an enzyme complex of the oxidase coenzyme Q with cytochrome c as the electron acceptor [24]. Mitochondrial membrane potential, reflecting the functional status of the mitochondrion, is found to be highly related to ROS levels [25, 26].

Cells have developed an antioxidant system to remove the excessive ROS. When the balance between the formation of ROS and antioxidant defense is damaged, oxidative stress occurs [18]. Oxidative stress can be caused by the following: (i) The level of endogenous and exogenous oxidants entering the body is increased, (ii) The reserve of antioxidants is consumed, (iii) The antioxidant enzymes are inactive, (iv) the production of antioxidant enzymes is reduced, and (v) Certain combination of the above two or more factors affects. Of course, redox imbalance may affect many other physiological and pathological processes [27]. Oxidative stress causes DNA damage, lipid peroxidation, protein modification, and other effects [28]. Oxidative stress is associated with numerous chronic pathological processes, including diabetes, cardiovascular diseases, atherosclerosis, thalassemia, cancers, chronic kidney disease, and neurodegenerative diseases such as Alzheimer's disease (AD) and Parkinson's disease (PD) [28–30]. Natural antioxidants derived from plants and other living organisms have been widely discussed as potential drugs in diseases caused by redox imbalance [31].

## 3. ROS and Antioxidant Pathways

Antioxidant defense is an important part for organisms to adapt to environmental stresses. Cells have developed different antioxidant responses to maintain redox homeostasis including endogenous antioxidant and redox-dependent transcriptional regulation pathways.

Antioxidant molecules are nucleophilic and react with oxidants, which are generally electrophiles. Glutathione (GSH), a ubiquitous low molecular weight thiol, is considered the most abundant endogenous antioxidant molecule [32]. GSH is a reduced peptide consisting of three-residues (*γ*-l-glutamyl-l-cysteinyl glycine), which can donate an electron to form oxidized GSSG. Alterations in the ratio of the redox pair 2GSH/GSSG towards a more oxidized status form the biochemical basis of targeting redox-sensitive cysteine residues in proteins. As an antioxidant, GSH removes ROS directly or indirectly and limits the lifetime of the oxidative signal [33]. GSH is also a substrate of several antioxidant enzymes. The indirect ROS-scavenging functions of GSH by revitalizing other antioxidant enzymes are also very important [34].

Multiple ROS sensors and pathways are triggered to converge in the regulation of transcription factors. So far, more than 20 redox-sensitive transcription factors have been reported [9, 35, 36]. These transcriptional factors induce the expression of multiple genes that are required for the detoxification and for the repair and maintenance of cellular homeostasis. In this review, we will discuss two well-studied ROS-sensitive transcriptional factors in detail as follows.

### 3.1. Nrf2 Pathway

**Figure 1:**
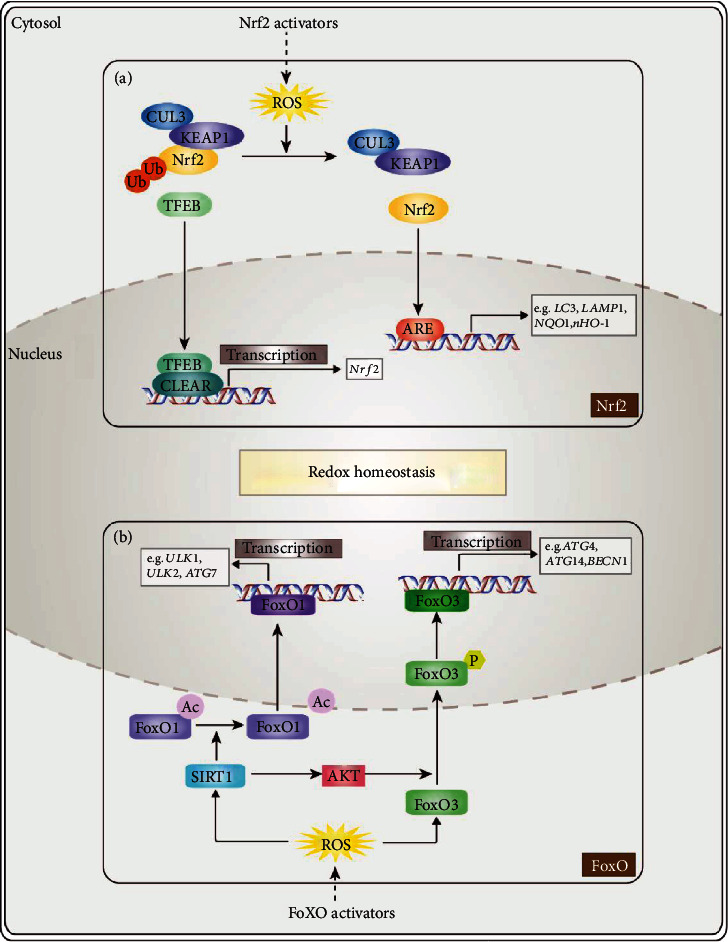
Schematic representation of redox-sensitive transcription factor-related antioxidant pathways. (a) Nrf2 pathway: when cells are exposed to ROS, Nrf2 dissociates from Keap1 and transfers into the nucleus, binding to ARE and regulating transcriptions of various antioxidant and lysosomal and autophagic genes [42]. Nrf2 activators, i.e., sulforaphane, show their protective effect against oxidative stress based on the Nrf2 signaling cascade [72]. (b) FoxO pathway: once activated by ROS, FoxO (mainly FoxO1 and FoxO3) transfers to the nucleus to initiate transcriptional activity [48]. Under oxidative stress, FoxO1 forms a complex with SIRT1 and deacetylates, resulting in preferential activation of autophagic and lysosomal genes [124]. Meanwhile, AKT, regulated by SIRT1, can phosphorylate FoxO3 proteins, thereby promoting the transcriptional activity of antioxidant-related genes. Resveratrol, gossypol acetic acid, etc., as FoxO activators, are reported to prevent chronic diseases by preventing oxidative stress and upregulate level of autophagy [125, 126].

Nuclear factor E2-related factor 2 (Nrf2), a redox-sensitive transcription factor, regulates multiple antioxidant gene expression and plays a crucial role in antioxidant pathways. Kelch-like ECH-associated protein 1 (Keap1) is the main regulator of Nrf2 [37]. Under normal conditions, Nrf2 binds to Keap1 and stays in the cytosol. Keap1 homodimer and cullin 3 (CUL3) combine to form a Keap1-CUL3 ubiquitin ligase complex, which catalyzes the polyubiquitination of Nrf2 to induce its degradation [38]. Under stress conditions, such as exposure to ROS, Nrf2 dissociates from Keap1 and transfers into the nucleus [9, 39]. Nrf2 then binds to the antioxidant response element (ARE) and increases the expression of downstream cytoprotective genes [40]. The Keap1/Nrf2/ARE system is the most crucial cytoprotective defense to oxidative stress (Figure 1) [41, 42].

### 3.2. FoxO Pathway

FoxOs are divergent members of the Fox/winged-helix transcription factor superfamily [43], which has various biological functions, including stopping the cell cycle at the G1-S and G2-M checkpoints, reduction of ROS, and repairing damaged DNA and apoptosis [7]. FoxO family members usually exist in the cytoplasm in an inactive form. Once activated, it will transfer to the nucleus to initiate transcriptional activity (Figure 1). FoxOs, composed of FoxO 1, 3, 4, and 6, coordinate gene expressions in cellular processes such as apoptosis and oxidative stress. For example, as a target of class III histone/protein deacetylase sirtuin 1 (SIRT1), FoxO1 forms a complex with SIRT1 under oxidative stress, resulting in activation of cell cycle arrest/anti-stress-related genes, thereby promoting cellular survival [44]. Several studies have shown that FoxOs and p53 have overlapping functions in cell cycle regulation and tumor suppression [45, 46]. In addition, p53 can directly target the FoxO3a gene, leading to an increase in FoxO3a in the nucleus, which causes apoptosis. FoxOs induce the expression of a number of autophagy-related genes (such as *Atg4*, *Atg7*, and *Atg14*), suggesting its role in autophagy regulation [47]. These evidences reveal that the FoxO-autophagy axis plays a crucial role in health and disease [48, 49].

### 3.3. The Effects of Single Antioxidant Treatment

Antioxidants including beta- carotene, lycopene, quercetin, resveratrol, and vitamin C have shown preventive effects in various diseases. However, poor biopharmaceutical properties and variable pharmacokinetics limit their application as therapeutic agents. For example, N-acetylcysteine (NAC), a powerful antioxidant that impacts GSH levels via cysteine, is approved by FDA [50, 51]. NAC is the precursor of GSH synthesis, which can scavenge free radicals and increase the content of GSH. Administration of NAC has shown protective effects against oxidative stress [52, 53]. However, the clinical effect of NAC is controversial [54, 55]. NAC's antioxidant effect lies in its ability to restore the cytosolic level of GSH, which is transported to mitochondria to exert its detoxification function. One example is the effect of NAC on Niemann-Pick disease type C (NPC). Reduced GSH levels have been detected in the liver of NPC mice with NAC treatment; however, the transport of GSH is delayed in NPC mouse hepatocytes [56, 57]. Thus, NAC is not effective in NPC treatment, although GSH increase in the cytoplasm. The transport of GSH to mitochondria is still defective. So far, single antioxidant treatment is not efficient, and more therapeutic approaches need to be explored.

## 4. ROS and Autophagy

Autophagy is a cellular self-eating phenomenon. It degrades and digests damaged, denatured, senescent, and loss-function cells, organelles, proteins, nucleic acids, and other biological macromolecules and participates in various processes such as biological development and growth. Autophagy is regulated by ROS and redox signaling including oxidized macromolecules and organelles, and mild oxidative stress [58]. Activated autophagy then removes damaged organelles and excessive ROS [59, 60]. ROS can oxidize cysteine residues of autophagy-associated proteins and modify their functions, facilitating the formation of the autophagosome [61] such as cysteine protease Atg4 [12].

ROS increase in the mitochondrial matrix can lead to mitochondrial damage and depolarization. The depolarized mitochondria are then fragmented, and PARK2 (mitochondrial E3 ubiquitin ligase) is recruited, leading to ubiquitination of damaged mitochondria [62], which are then phagocytosed by LC3-positive autophagosomes and directed to lysosomes for degradation [63]. This process is called mitophagy. Under starvation, mitophagy is triggered by mitochondrial ROS to remove damaged mitochondria and other organelles [14]. In turn, damaged mitochondria will produce more ROS. Mitochondrial dysfunction has been considered as a key factor in neurodegenerative diseases, which contains a high level of ROS in the brain [64]. Thus, autophagy promotion has been considered as a potential treatment for neurodegenerative diseases.

### 4.1. TFEB as a Drug Target

**Figure 2:**
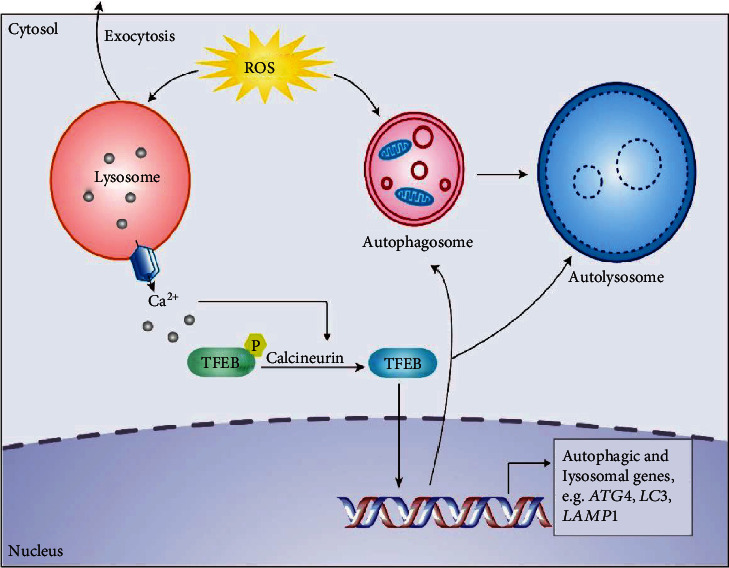
ROS regulates TFEB-dependent autophagy promotion. Lysosomes are activated by mitochondrial ROS, followed by lysosomal Ca^2+^ release and calcineurin activation. Calcineurin bound to Ca^2+^ dephosphorylates TFEB. Then, nuclear-localized TFEB causes the transcription of a series of genes, including autophagy induction, autophagosome biogenesis, lysosomal biogenesis, and autolysosome biogenesis [60, 69]. Autophagy is enhanced to promote the removal of damaged mitochondria and excess ROS [127]. Among them, a low level of oxidative stress will stimulate lysosomal exocytosis, but at a high level, it will inhibit lysosomal exocytosis [128].

Transcription factor EB (TFEB), a master regulator of the autophagic and lysosomal biogenesis, acts as a critical mediator of the cellular response to stress (Figure 2) [65, 66]. TFEB binds to the “coordinated lysosomal expression and regulation (CLEAR)” element located in the promoter region of many lysosomal and autophagic genes [67]. TFEB is responsive to multiple types of intracellular stress including mitochondrial damage and oxidative stress [68]. Increased ROS levels can lead to activation of transient receptor potential mucolipin 1 and lysosomal calcium release, which induces nuclear translocation of TFEB and then promotes autophagic and lysosomal biogenesis [69]. TFEB activity is controlled by its phosphorylation status. In nutrient-rich conditions, TFEB is phosphorylated and retained in the cytoplasm. Upon starvation, TFEB is dephosphorylated and translocated from the cytoplasm to the nucleus, regulating the expression of target genes [70]. According to its important role in promoting autophagy and lysosome, TFEB has become an important therapeutic target for diseases involving excess ROS and autophagy dysfunction, such as AD, PD, and atherosclerosis (see Figure 2 for details about TFEB and autophagy). Recently, several TFEB agonists have been identified and preclinical or clinical trials are applied [71, 72].

### 4.2. The Effects of Autophagy Activators

Mammalian target of rapamycin (mTOR), a critical nutrient sensor, can regulate TFEB [73]. Under nutrient-rich conditions, mTOR phosphorylates TFEB on serine residues of S142 and S211 and phosphorylated TFEB is retained in the cytosol [74]. Upon starvation, mTOR is inhibited and TFEB is activated and translocated into the nucleus [75]. Torin1, 3,4-dimethoxychalcone, fisetin, and rapamycin are mTOR inhibitors [76–79].

Rapamycin has been shown to upregulate autophagy in cell models, fruit fly models, and mouse models of neurodegenerative diseases, respectively [80–83]. In the model of Huntington's disease (HD), rapamycin treatment can simultaneously reduce the soluble mutant huntingtin and the aggregation products of the protein, thereby protecting the cells from damage. A similar situation also appears in the PD model [83]. However, in early trials, high-dose treatment of rapamycin causes frequent side effects including slow wound healing and hyperlipidemia [84]. In addition, high-dose or long-term use of rapamycin in patients causes severe infection, hemolytic uremic syndrome, cancer, leukopenia, bone atrophy, and even noninfectious interstitial pneumonia [85].

Lithium can negatively regulate the activity of GSK-3*β*, leading to the stimulation of mTOR kinase and the inhibition of autophagy. Recently, the combined use of lithium and rapamycin is found to be much more effective than rapamycin alone [86]. In addition, due to the limited absorption of rapamycin, its derivatives have been developed such as temsirolimus, everolimus, and lidformolimus [87, 88].

## 5. ROS as a Link to Connect Autophagy and Antioxidant Pathways

Many diseases such as NPC are associated with both oxidative stress and autophagy dysfunction. So far, none of single therapy against one target is shown to be effective. Thus, the dual-target therapeutic drugs will shed new light on the future directions. A number of natural compounds are identified to reduce oxidative stress and promote autophagy. Moreover, preclinical and clinical studies have shown that natural compounds, such as resveratrol, have therapeutic potential in several diseases including diabetes, aging, neuropathy, cardiovascular diseases, and cancer [89].

**Figure 3:**
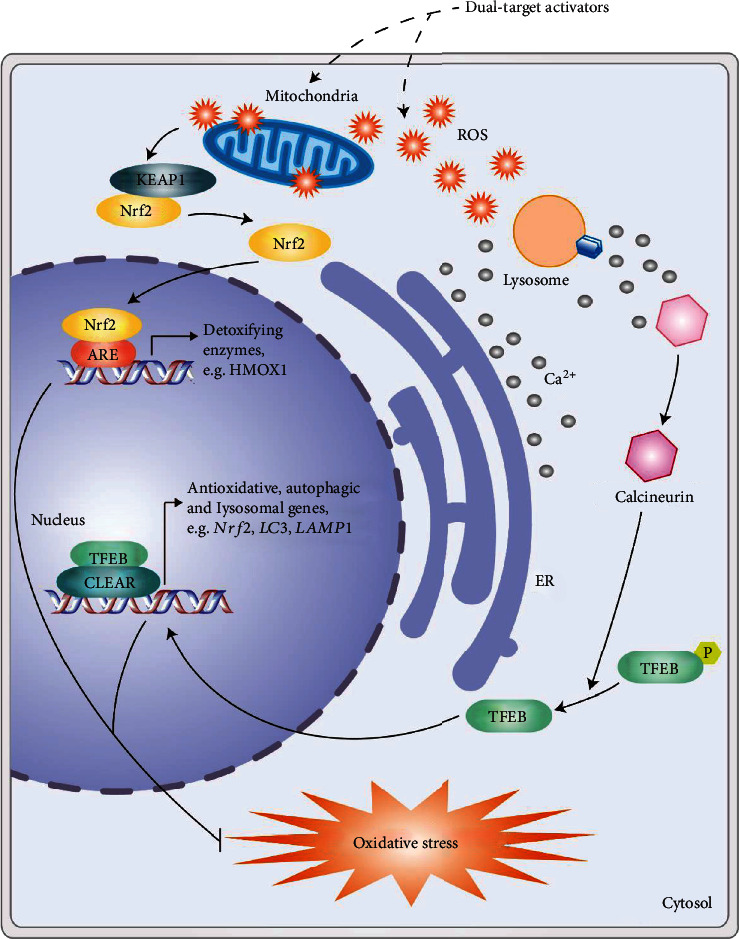
Dual-activating (antioxidant and autophagy) pathways. Dual-target activators such as sulforaphane induce a low level of ROS to activate the Nrf2-dependent antioxidant pathway and TFEB-dependent lysosomal biogenesis and autophagy, thereby helping to remove excess ROS [72]. A working model to illustrate the role of Nrf2/TFEB in sulforaphane-mediated enhancement of autophagic and lysosomal function. Sulforaphane (for example, through mitochondria and other sources) stimulates low level of ROS, which activates the Nrf2 pathway and the release of Ca^2+^. Ca^2+^-bound calcineurin dephosphorylates TFEB, causing TFEB nuclear translocation [129]. Nuclear Nrf2/TFEB then promotes the transcription of a unique set of genes related to detoxifying enzymes, autophagy induction, and autophagic and lysosomal biogenesis [130]. Subsequently, the cells are promoted to remove damaged mitochondria and excess ROS (the figure is adapted from Li et al. [72]).

Sulforaphane, an Nrf2 activator enriched in cruciferous vegetables, has several biological activities such as reduction of oxidative stress and inflammation in several diseases including AD, sclerosis, and traumatic brain injury [90–93]. Recently, we found that sulforaphane is also a TFEB agonist [72]. Sulforaphane activates TFEB via stimulating low level of ROS, then inducing the expression of genes required for lysosomal biogenesis, autophagosome formation, and detoxification. A genetic interaction between Nrf2 and TFEB is also identified. Altogether, sulforaphane is a dual-target candidate for diseases with excessive ROS and autophagy dysfunction (Figure 3). Other phytochemicals with dual-target therapeutic effects are also summarized as follows (Table 1).

Flavonoids, a family of natural products enriched in fruits, have biological activities including anticancer, antiproliferation, antioxidant, and anti-inflammation via regulation of the cell cycle, induction of apoptosis, and inhibition of extracellular protein kinase phosphorylation [94]. Flavonoids have therapeutic effects on several diseases such as diabetes, cancer, and cardiovascular diseases [95]. Kaempferol, a flavonoid, induces autophagic cell death in gastric cancer cells through epigenetic changes [96]. Quercetin provides neuroprotection by stimulating Nrf2-ARE antioxidant defenses and inducing autophagy induced via SIRT1 [97]. Modifications of flavonoids, such as hydroxylation, glycosylation, methylation, and acylation, have been shown to improve their biological activity [98–101].

Isoflavones are a variety of secondary metabolites mainly distributed in legumes [102]. They regulate the expression of antioxidant proteins and induce autophagy, thus eliminating the damaged or dysfunctional organellesand playing a cytoprotective role in maintaining cell homeostasis. Genistein, a soy-derived isoflavonoid with antitumor activity, involves the regulation of antioxidant enzymes and the expression of apoptotic signals, leading to the progression of cell apoptosis and autophagy [103].

Resveratrol, a ROS scavenger extracted from red grape skins and peas [104, 105], has many activities including antiaging and anticancer [106–110]. Studies have shown that resveratrol activates SIRT1, which may rely on the upstream of calmodulin kinase II to activate the AMPK-dependent increase in the ratio of NAD/NADH, thereby inducing SIRT1 activity. Resveratrol can promote p53 deacetylation and downregulate Akt phosphorylation, then increasing SIRT1 expression [111, 112]. Resveratrol inhibitscancer cell growth through autophagic initiation. Resveratrol also increases the chemotherapeutic efficiency of gemcitabine via Nrf2 signaling [113]. In addition, the anticancer activity of resveratrol is related to the activation of FoxOs. Resveratrol inhibits PI3K/Akt phosphorylation, resulting in a decrease in FoxO3 phosphorylation and an increase in FoxO3 nuclear transport, DNA binding affinity, and transcriptional activity [114]. In clinical trials, resveratrol can alleviate clinical parameters of cardiovascular diseases [115–117].

Curcumin, a major active component of turmeric (Curcuma longa, L.), has anticancer, anti-inflammatory, and antioxidant effects and has been applied to cancer, atherosclerosis, and neurodegenerative diseases [118–120]. Low dose of curcumin induces adaptive oxidative stress responses, while high dose of curcumin induces acute responses such as autophagy and mitochondrial destabilization [121]. This phenomenon is often referred to as hormesis. However, curcumin has poor bioavailability. Curcumin analogs, such as the neoketene curcumin, have stronger clearance capabilities and become potential drugs under different pathological conditions [122].

## 6. Conclusions

Excessive ROS have been implicated in many diseases including cancer, neurodegenerative diseases and aging. Low/mild levels of ROS have been identified as important cellular signals, which can induce autophagy and antioxidant pathways under both physiological and pathological conditions. Increasing evidence suggests that there may be an important link ROS, antioxidant pathways, and autophagy. The detailed molecular mechanism underlying this linkage remains elusive. Antioxidants or autophagy activator alone is not ideal treatment for diseases characterized by both oxidative stress and autophagy dysfunction. Natural compounds with dual targeting of antioxidant and autophagy such as sulforaphane could be the potential therapeutic drug and direction for future research.

## Figures and Tables

**Table 1:** Summary of phytochemicals related to ROS scavenging.

Phytochemicals	Sources	Main properties
Sulforaphane	Cruciferous plants such as broccoli [72]	Induce the expression of genes required for lysosomal biogenesis, promote autophagic flux, and induce TFEB nuclear translocation [72]
Flavonoids	Citrus [95], rutin, and so on	Antioxidant, suppression of carcinogenesis [94], and anti-inflammation [95]
Isoflavones	Legumes from the family *Fabaceae* [102], namely, soybean	Antioxidant, induction of autophagy, antitumor effect [103], and anti-inflammation [123]
Resveratrol	Red grape skins, peas, and so on	Antiaging [109], anticancer [106], anti-inflammation, and prevention of cardiovascular diseases
Curcumin	Rhizomes of some plants such as *Zingiberaceae*	Anticancer, anti-inflammatory, and antioxidant [118]

## Data Availability

Not applicable-no new data generated in this study.

## Abbreviations

AD: Alzheimer's disease
ARE: Antioxidant response element
C-I: Complex I
CLEAR: Coordinated lysosomal expression and regulation
CUL3: Cullin 3
FoxO: Forkhead box, subgroup O
GSH: Glutathione
HD: Huntington's disease
IKK: I*κ*B kinase
Keap1: Kelch-like ECH-associated protein 1
mTOR: Mammalian target of rapamycin
NAC: N-Acetylcysteine
NPC: Niemann-Pick disease type C
Nrf2: Nuclear factor E2-related factor 2
PD: Parkinson's disease
ROS: Reactive oxygen species
SIRT1: Sirtuin 1
TFEB: Transcription factor EB.
